# Infection prevention and control of the Ebola outbreak in Liberia, 2014–2015: key challenges and successes

**DOI:** 10.1186/s12916-015-0548-4

**Published:** 2016-01-05

**Authors:** Catherine Cooper, Dale Fisher, Neil Gupta, Rose MaCauley, Carmem L. Pessoa-Silva

**Affiliations:** Ministry of Health and Social Welfare, PO Box 10–9009, 1000 Monrovia 10, Liberia; Division of Infectious Diseases, University Medicine Cluster, National University Hospital, Singapore, Singapore; Department of Medicine, Yong Loo Lin School of Medicine, National University of Singapore, Singapore, Singapore; Division of Healthcare Quality Promotion, Centers for Disease Control and Prevention, Atlanta, GA 30333 USA; John Snow, Inc., Boston, MA 02210-1211 USA; Pandemic and Epidemic Department, World Health Organization, Geneva, Switzerland

**Keywords:** Liberia, Ebola, Infection prevention and control, Outbreak response, GOARN

## Abstract

Prior to the 2014–2015 Ebola outbreak, infection prevention and control (IPC) activities in Liberian healthcare facilities were basic. There was no national IPC guidance, nor dedicated staff at any level of government or healthcare facility (HCF) to ensure the implementation of best practices. Efforts to improve IPC early in the outbreak were *ad hoc* and messaging was inconsistent. In September 2014, at the height of the outbreak, the national IPC Task Force was established with a Ministry of Health (MoH) mandate to coordinate IPC response activities. A steering group of the Task Force, including representatives of the World Health Organization (WHO) and the United States Centers for Disease Control and Prevention (CDC), supported MoH leadership in implementing standardized messaging and IPC training for the health workforce. This structure, and the activities implemented under this structure, played a crucial role in the implementation of IPC practices and successful containment of the outbreak. Moving forward, a nationwide culture of IPC needs to be maintained through this governance structure in Liberia’s health system to prevent and respond to future outbreaks.

## Background

The outbreak of Ebola virus disease (EVD) in West Africa, unprecedented in both size and duration, appears in its terminal stages as of the last quarter of 2015. Historically, Ebola outbreaks typically last no more than 3–4 months from the time of identification and involve, at a maximum, hundreds of patients. This outbreak, affecting Guinea, Sierra Leone and Liberia, resulted in greater than 28,000 reported cases, with over a third of these from Liberia [1].

The first cases in the region were identified in Guinea in March 2014 and subsequently spread across the border to Liberia when a patient, infected in Guinea, presented for treatment at Foya Borma Hospital, along the border in Lofa County. This first wave of transmission resulted in six EVD infections and demonstrated a national vulnerability when one of these patients travelled to Margibi County, 400 km away [2]. A second wave occurred when a patient presented to the same hospital in Lofa County on 23 May 2014, after travelling from Sierra Leone. From this time, transmission intensified with all counties soon affected [3].

Early in the outbreak, several clusters of EVD were reported in healthcare facilities throughout the country [4, 5]. These clusters occurred in part because of poor knowledge and adherence to basic infection prevention and control (IPC) practices, and contributed to EVD transmission among patients and healthcare workers (HCWs) within the healthcare facility and surrounding communities. By March 2015, 288 HCW infections had been reported in Liberia [6]. As stories spread about the risk of healthcare-associated EVD, many HCWs were afraid to return to work. This resulted in a rapid breakdown of essential health services, with consequent ramifications for endemic infectious diseases (e.g. measles, malaria, HIV, tuberculosis) and non-communicable disease-related care, such as diabetes and maternal health [7].

Early outbreak response efforts demanded a new level of intensity once the scale and further potential for spread was realized. The incidence of cases began falling in October 2014 and on 9 May 2015, Liberia was declared Ebola-free by the World Health Organization (WHO). Although another cluster was identified on 29 June 2015, this was quickly contained [8] and Liberia was again declared free of Ebola transmission on 3 September 2015. Liberia ultimately documented over 10,000 cases, including more than 4,000 deaths.

The purpose of this commentary is to describe specific efforts critical to the success of the IPC response in Liberia. Due to the rapid nature of the response, some of the information reported is based upon unpublished data or personal observations.

## Discussion

### Health service delivery and healthcare workers during the outbreak

By September 2014, the number of Ebola Treatment Unit (ETU) beds needed critically exceeded the number of beds available. Because ETUs were primarily being established in response to local clusters, new cases would frequently present in areas distant from this structured care. This logistic difficulty of providing appropriately located ETU beds in adequate numbers was a challenge for those managing the response as patients wanted care close to home. Patients with symptoms of Ebola were presenting to clinics, healthcare centers and hospitals for care; therefore, HCWs all over the country had to enhance triage practices to facilitate the urgent identification and isolation of suspected cases, and provide potentially lifesaving care until they could be safely transferred to an ETU. With many HCFs closed and patients turned away from ETUs that were full, community leaders requested IPC guidance and support for community-based care.

### IPC activities early in the outbreak

Initial engagement by international and local IPC specialists with Liberian HCWs primarily focused on dispelling myths about the origins of the disease: Ebola was real; it was an infectious disease; and lives could be saved with supportive care. HCWs needed the reassurance that they could safely provide care with the right personal protective equipment (PPE) and training. In the early stage of the outbreak, IPC practices in non-ETU healthcare facilities in Liberia were limited. Although several organizations provided some IPC training, there was no national strategy or appropriate oversight at the national, county or facility level. As a result, messaging was inconsistent and coordination *ad hoc*. As the outbreak escalated it became clear that variations in messages from different organizations were confusing. Additionally, HCWs were being trained in the absence of adequate supplies. This fragmented, poorly coordinated system for IPC support underscored the need for improved oversight and supervision nationally [9].

### Establishing the national IPC Task Force

**Fig. 1:**
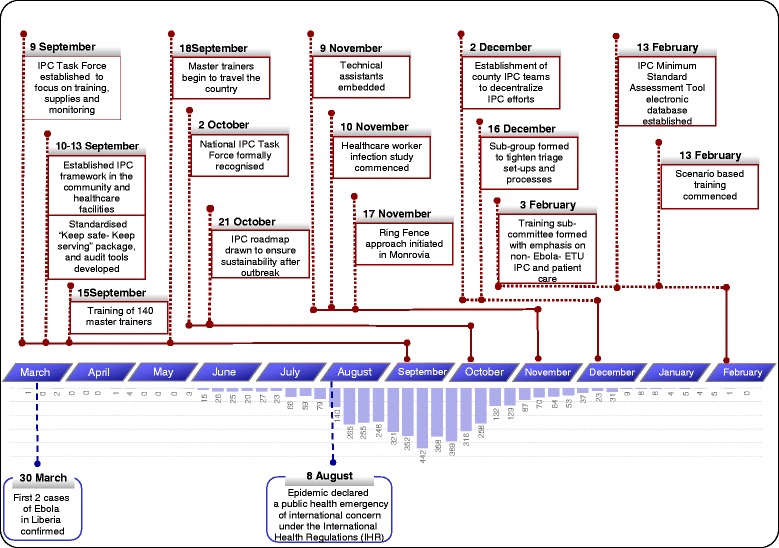
Timeline of the national IPC Task Force in Liberia: September 2014 to March 2015. The National IPC Task Force was established at the peak of the outbreak in September 2014. Specific efforts in the subsequent 6 months are highlighted in the figure and aligned to the epidemic status at the time. The numbers in the bars represent the weekly number of confirmed cases of EVD in Liberia

In September 2014, with transmission continuing unchecked, the national IPC Task Force was established. Chaired by the Ministry of Health (MoH) and supported by international partners and local non-governmental organizations (NGOs), the Task Force rapidly developed a national policy and guidelines for IPC practices in general healthcare facilities. Leveraging partnerships with organizations on the ground, the Task Force assumed the role as the coordinating body for IPC activities in Liberia, standardizing messages, overseeing resource needs (especially PPE), establishing IPC standards, and providing tools to assess compliance with these standards (Fig. 1). The Task Force also provided advice on enhancing prevention measures in the community, such as improving hygiene and infection prevention measures in congregate settings [10]. The national IPC Task Force succeeded in its primary goal, to speak as one voice through an all-inclusive principle (community-based, faith-based organizations, national and international NGOs, etc.). Through technical oversight, regular communication and strong partner relationships, the Task Force helped drive several initiatives crucial to the response.

### The Keep Safe, Keep Serving (KSKS) programme

Over 2 weeks, a technical working group of the Task Force developed numerous guidelines, posters and training materials adapted to the specific needs of non-ETUs in Liberia at the time. The most significant issue reported by HCWs was the need for consistent and clear guidance for PPE. Based on fear for their personal safety, many HCWs felt conventional PPE was inadequate. There were overt differences between what was worn in ETUs and what was recommended by the WHO and Centers for Disease Control and Prevention (CDC).

To enhance protection against EVD during the outbreak, instill confidence in Liberian HCWs and facilitate rapid uptake of new guidance around the country, a simple two-tier, risk-based PPE protocol was issued. All HCWs were recommended to wear “basic PPE” (consisting of face shield, gown and gloves) for all low-risk clinical activities. In contrast, “enhanced PPE” (basic PPE plus mask, head cover or hood, apron and a second pair of gloves) was recommended for all HCWs providing care to suspected or confirmed EVD patients, or conducting other high-risk activities. Coveralls were an option in the enhanced PPE guideline.

The work of the national IPC Task Force, its steering committee and technical working group provided for a system that would ensure a safe working environment for all HCWs. The workflows were designed to facilitate the rapid identification, isolation and care of patients with EVD until they could be transferred to an ETU. The programme branded “Keep Safe, Keep Serving” (KSKS) also enabled restoration of preexisting health services. The group had to provide recommendations for situations with no evidence or clinical precedents, such as care of Ebola patients in the community when ETU bed capacity was exceeded [11].

By the end of September 2014, 120 healthcare workers trained in the KSKS package travelled across the country to train regional healthcare workers to implement these guidelines. Based on MoH policy, all partners had to use the standardized KSKS documents accessible via a shared internet folder. This concerted approach allowed for considerable reach throughout the health system; by December 2014, over 4,000 HCWs had been trained in the KSKS curriculum and these posters and training materials were evident in facilities throughout the country.

Within months of establishing its “flagship” KSKS programme, the national IPC Task Force was increasingly recognized for its authority and influence in Liberia. The Task Force began to expand its scope of activities to facilitate the implementation of best IPC practices. To help translate KSKS didactic sessions into practice, role play simulations testing triage, isolation, PPE use and environmental cleaning were introduced to complement training.

The Task Force also coordinated with the relevant logisticians to help inform the national supply chain and facilitate distribution of PPE to all non-ETU healthcare facilities so that KSKS policies could be implemented. To enhance local capacity building, the Task Force initially trained and deployed 21 specialists in IPC, termed technical assistants (TAs), into the healthcare teams of 14 counties to provide county-level IPC support. Among other responsibilities, these embedded TAs worked with county healthcare teams and partners to establish county IPC committees, identify and train facility IPC specialists, and ramp up triage and isolation practices through intensified training and establishment of purpose-built temporary structures.

### The sector approach

In January 2015, the “sector approach” was introduced to intensify response efforts in areas with continued active transmission in Montserrado County, the most populous county in Liberia which contains the capital city Monrovia [12]. Montserrado County was divided into four geographic sectors, each with its own team. Each team focused primarily on healthcare facility readiness, with an emphasis on triage. Although the national IPC Task Force continued to set priorities and establish minimum standards, the implementation and monitoring of these standards in Montserrado was delegated to sector teams. These intensified efforts, implemented in a “ring approach”, helped Liberia approach its goal of “getting to zero” after identification of the cluster of 22 EVD infections near St Paul Bridge in Monrovia in February 2015 [13].

### Facing the challenges

The IPC component of the outbreak response had come a long way. Early in the outbreak, the absence of IPC practices contributed to a fertile setting for Ebola virus transmission. Six months into the outbreak genuine strategic efforts were initiated, which were likely crucial in ending the outbreak of EVD in Liberia. In 2015, the few cases in March and the recurrence of the outbreak in June and November were met with robust IPC responses suggestive of a sustained capacity.

Practicing IPC in the absence of PPE is very difficult, but additional logistic issues were also routinely encountered. Poor equipment and communications, lack of running water and reliable power, inadequate cleaning supplies and often lack of pay to HCWs all increased the challenge. The Liberian workforce needed international technical support and this likewise represented a constant challenge.

In Liberia, there were well over 30 organizations contributing to the IPC response, including international partners and local NGOs (Table 1). Of the NGOs already operating in country few had technical expertise in IPC, thus additional assistance was required. However, lengthy deployment of IPC experts was often difficult and resulted in high turnover with variability in the training and expertise of international staff. At times this compromised the availability of quality and consistent support for the Liberian workforce. The risk of poor continuity was alleviated by the sustained authority of the IPC Task Force and the KSKS programme. Balancing the expertise of international IPC professionals with the experience of local organizations was crucial to the response. Technical and interpersonal skills, as well as the ability to compromise and adapt, were vital.

**Table 1:** Some organizations involved in the IPC response in Liberia (in alphabetical order)

Accel Partners	Action Contre La Faim	African Union
AmeriCares	American Refugees Committee	Centers for Disease Control and Prevention
Christian Health Association of Liberia	Community Health and Education Social Services, Liberia	Catholic Relief Services
Diakonie Katastrophenhilfe	eHealth Africa	EQUIP Liberia
International Committee of the Red Cross	International Media Support International Medical Corps	International Medical Corps
International Organization for Migration	Johns Hopkins Program for International Education in Gynecology and Obstetrics	John Snow, Inc.
Last Mile Health	Médecins du Monde	MENTOR Initiative
Swedish Civil Contingencies Agency	Médecins Sans Frontières	Open Society Foundations
Oxfam	Partners In Health	Project Concern International
Save the Children	United Nations Population Fund	United Nations Children’s Fund
United States Agency for International Development	World Food Programme	World Health Organization
U Foundation		

## Conclusions

No single organization could respond as comprehensively as was required during the 2014–2015 Ebola outbreak. IPC cannot be implemented in the absence of complementary outbreak response teams managing epidemiology, surveillance, laboratory, clinical cases, funerals, logistics and security, social mobilization and health education, psychosocial support, and other key functions. The WHO’s Global Outbreak Alert and Response Network (GOARN) deployed over 900 technical experts to West Africa for this outbreak, and is well placed to train and ultimately call upon a workforce, especially in collaboration with its largest partners. To more rapidly and efficiently respond to future outbreaks the professional development of, and prospective commitment from, adequately trained experts from current and future partners should be obtained.

In Liberia, poor IPC capacity was the primary driver of EVD transmission within the health system [6]. While the activities of the national IPC Task Force helped address weaknesses in IPC during the outbreak, these improvements need to be integrated into all aspects of healthcare delivery and training of the workforce going forward. To be successful, the national Keep Safe, Keep Serving programme has to be restructured from its Ebola-specific focus to emphasize the importance of standard precautions in everyday practice. Healthcare workers need to receive continuous training on risk-based practices for high priority diseases in the region, and dedicated IPC specialists must be present at the national, county and facility level to ensure adherence to these essential quality practices. In addition, better coordination with the national supply chain and investments in basic infrastructure for power, water and sanitation are necessary to create a safe environment for delivery of health services. Embracing these practices will help institutionalize a culture of IPC in the nation’s health system, and hopefully leave Liberia with something positive from this national tragedy.
